# Person-centered care for common mental disorders in Ontario’s primary care patient-centered medical homes: a qualitative study of provider perspectives

**DOI:** 10.1186/s12875-024-02519-w

**Published:** 2024-08-02

**Authors:** Matthew Menear, Rachelle Ashcroft, Simone Dahrouge, Jose Silveira, Jocelyn Booton, Monica Emode, Kwame McKenzie

**Affiliations:** 1 Department of Family Medicine and Emergency Medicine, Université Laval, Quebec City, Canada; 2 VITAM Centre de recherche en santé durable, Quebec City, Canada; 3 Factor-Inwentash Faculty of Social Work, University of Toronto, Toronto, Canada; 4 Department of Family Medicine, University of Ottawa, Ottawa, Canada; 5 Department of Psychiatry, University of Toronto, Toronto, Canada; 6 School of Population and Public Health, University of British Columbia, Vancouver, Canada; 7 Wellesley Institute, Toronto, Canada

**Keywords:** Primary care, Mental health, Person-centered care, Common mental disorders, Patient-centered Medical Home, Canada

## Abstract

**Background:**

For more than a decade, the Patient-Centered Medical Home model has been a guiding vision for the modernization of primary care systems. In Canada, Ontario’s Family Health Teams (FHTs) were designed in the mid-2000s with the medical home model in mind. These primary care clinics aim to provide accessible, comprehensive, and person-centered primary care services to communities across Ontario. Their services typically include mental health care for people experiencing common mental disorders, such as depression and anxiety disorders. It remains unclear, however, whether the mental health care delivered within FHTs is consistent with person-centered care approaches. In the current study, we aimed to explore the perspectives of FHT providers on the care delivered to people with common mental disorders to determine whether, and to what extent, they believed this care was person-centered.

**Methods:**

We conducted a qualitative grounded theory study involving interviews with 65 health professionals and administrators from 18 FHTs across Ontario. Transcripts were coded using a three-step process of initial, focused, and axial coding that mixed inductive and deductive approaches informed by sensitizing concepts on person-centeredness.

**Results:**

Practices and challenges associated with the delivery of mental health care in a person-centered way were captured by several themes regrouped into five domains: (1) patient as unique person, (2) patient-provider relationship, (3) sharing power and responsibility, (4) connecting to family and community, and (5) creating person-centered care environments. FHT providers perceived that they delivered person-centered care by delivering mental health care that was responsive, flexible, and consistent with biopsychosocial approaches. They emphasized the importance of creating long-lasting relationships with patients grounded in empathy and trust. Their challenges included being able to ensure continuity of care, adequately prioritizing patients’ mental health issues, and meaningfully engaging patients and families as partners in care.

**Conclusions:**

Our findings suggest that FHT providers have adopted a range of person-centered practices for people with common mental disorders. However, greater attention to practices such as shared decision making, supporting self-management, and involving families in care would strengthen person-centeredness and bring teams closer to the Patient-Centered Medical Home vision.

**Supplementary Information:**

The online version contains supplementary material available at 10.1186/s12875-024-02519-w.

## Background

Primary care services are the foundation of health care systems. Ensuring access to high-quality primary care is critical to meeting population health needs, managing health care costs, and promoting population health [1, 2]. Person-centered care is a core component of high-quality primary care and is broadly defined as a relationship-based approach, oriented to the whole person, that recognizes service users and their families as core members of the care team [3]. In a person-centered approach, care is organized around the unique and comprehensive needs of people rather than individual diseases [4]. It extends beyond clinical encounters and involves understanding patients as people, their families, their social world, and the communities in which they work and live [1, 2, 4]. Despite widespread support for person-centered care, primary care practitioners do not always have the capacity or resources to apply this approach in routine practice [1, 5–7]. The Patient-Centered Medical Home model has thus been advanced as an organizing concept for modern primary care systems aligned with the principles of patient-centeredness. This model promotes an interdisciplinary team structure and the delivery of holistic, evidence-based care that is easily accessible, coordinated across providers and settings, and respectful of diverse needs, cultures, and values [8].

Originally developed in the U.S. in the 2000s, the Patient-Centered Medical Home model has since spread to many other countries, including Canada. Ontario’s Family Health Teams (FHTs) are one of Canada’s best examples of the Patient-Centered Medical Home in action. FHTs were first introduced in 2005 and created to improve access to broad, person-centered primary health care services to communities across Ontario [9]. Today, 187 FHTs serve over 3 million people, or approximately 22% of the province’s population [10]. In FHTs, family physicians work alongside other health professionals in a team-based approach to provide continuous and coordinated care to their communities. Community needs can influence the services of individual sites but overall the FHT model aligns well with the principles of the Patient-Centered Medical Home [11] and attributes of the Patient’s Medical Home model promoted by the College of Family Physicians of Canada [12].

Among the defining features of these medical home models are their emphasis on whole-person care and the seamless integration of services within teams and the broader health system [11–13]. This includes a capacity to meet the needs of people experiencing mental health concerns. Indeed, numerous reports have emphasized the importance of integrated mental health care as a central and necessary component of the medical home model [14–17]. In Ontario, most FHTs include professionals that focus on mental health care, such as social workers, mental health counsellors, psychologists, and general mental health workers [18]. However, the delivery of high-quality, integrated mental health care in primary care remains a challenge, even in settings like FHTs that are aligned to the Patient-Centered Medical Home model [19–22].

An important challenge for FHTs and other clinics adhering to the medical home model is ensuring that the mental health services they do provide are truly person-centered. It is not uncommon for people with common mental disorders (e.g., depression, anxiety disorders) to report negative care experiences, such as encountering unsupportive or paternalistic attitudes, experiencing poor communication with providers, or having limited involvement in treatment decisions [23–25]. While the medical home model may be the appropriate vision for how care should be provided, investigations into the person-centeredness of mental health care in medical homes specifically and even primary care more generally have been sparse [6]. Authors have notably insisted on the need for more studies exploring the perspectives of those people directly involved in providing or receiving integrated mental health care [6, 26–28]. We found only one qualitative study examining providers’ perspectives on the person-centeredness of mental health care in diverse clinical settings (including primary care) in the Veterans Administration system [6], and no studies on this topic from Canada. Most other qualitative studies on the quality of care for mental disorders in primary care have instead focused on the technical aspects of managing these conditions (e.g., diagnosing, treating) [29–32] or have examined experiences of care broadly without a specific focus on patient-centeredness [26, 33–36].

To address these knowledge gaps, we aimed to explore the perspectives of FHT providers regarding their experiences providing care for common mental disorders to determine whether, and to what extent, they believed this care was person-centered.

To address these knowledge gaps, we aimed to explore the perspectives of FHT providers on the person-centeredness of care delivered within FHTs to people with common mental disorders. Our research question was: What are the experiences of FHT providers regarding the delivery of person-centered care to people with common mental disorders and what are the challenges they encounter delivering person-centered mental health care? We hoped that a deeper understanding of these providers’ experiences would enable us to identify potential areas to strengthen the quality and person-centeredness of mental health care in FHTs.

## Methods

### Study design

This study was part of a larger, 4-year qualitative study investigating the influence of financial and non-financial incentives on the quality of mental health care in Ontario’s FHTs [20, 37]. This study relied on a constructivist grounded theory methodology that informed our study sampling, data collection, and data analysis [38]. Charmaz’s constructivist approach was considered appropriate given our interest in using an inductive approach to grounded theory that could be informed by sensitizing concepts from the literature on quality of care. Indeed, several concepts drawn from previous frameworks [39–41] and reviews [42–44] were considered useful in our analyses of data on person-centeredness. Here, we present the findings from our in-depth analysis of the data on person-centeredness from the larger parent study. The reporting of our findings is consistent with COREQ reporting standards [45].

This study received Research Ethics Board Approval from the University of Waterloo, University of Toronto, the Centre for Addiction and Mental Health (CAMH), Bruyère Continuing Care, St. Joseph’s Health Centre/Unity Health Toronto, and Université Laval.

### Sampling and recruitment

Study sampling was conducted in two phases. First, we used purposive sampling to select a diverse sample of FHTs that varied in their geographic location (urban/rural), team size, and team composition, using information from the Ontario ministry of health. Second, we used a combination of maximum variation and theoretical sampling approaches to sample participants from the FHTs. Any provider within the FHT was eligible to participate, including executive directors, family physicians, nurse practitioners, nurses, social workers, mental health workers, and other professionals. Psychiatrists working in a shared care model that delivered care with FHTs were also eligible for inclusion. The larger parent study also featured interviews with several policymakers and community providers in Ontario but data from these interviews was not considered for the current study. We sent invitational letters by email to the executive directors or medical leaders of the FHT, who were invited to share information about the study with their team. Providers interested in participating in the study were invited to contact the lead investigator (RA) and/or the research coordinator by email or phone. When additional recruitment at a site was deemed necessary, executive directors or medical leaders facilitated recruitment by identifying potential participants with specific profiles (e.g., family physicians, mental health workers, etc.) and helping to connect them to the research team, who then proceeded to inform them about the study.

### Data collection

Data was collected through individual, semi-structured interviews conducted at the FHT sites (e.g., participants’ offices). The interviews were conducted by the study’s lead investigator (RA), an experienced qualitative researcher and professor with a background in social work. All participants were informed about the study’s goals and provided written consent to participate prior to their interview. Interview guides were used to structure the interviews and included questions about providers’ role and experience at the FHT, the mental health care delivered at the FHT, and their experiences providing care to people common mental disorders. The interview guides included prompts specific to person-centeredness, including providers’ person-centered practices (e.g., involving patients in care, supporting self-management) and the challenges and facilitators of this approach to care (see Appendix). Interviews had an average duration of just over 60 min (range 27–105 min). Repeat interviews to clarify previously collected information and capture additional data for analyses occurred with 14 participants (their duration was similar to the initial interviews). Interviews were audio-recorded, transcribed verbatim, and reviewed for accuracy immediately following the interview. The lead investigator also used memo-writing immediately after each interview to record her impressions of the interview and notes to consider during the analysis [38].

### Data analysis

We relied on an iterative approach to analysis in which data collection and data analysis occurred simultaneously [38, 46]. Analysis began immediately following the transcription of each interview. The coding process involved three steps: initial, focused, and axial coding [38, 46]. Initial coding involved line-by-line open coding of interviews to tie concepts to blocks of raw data. Focused coding then entailed a constant comparative approach to reconcile codes and identify those that appeared frequently or were considered more significant. Focused codes were often labelled with gerunds, which builds action into the data and helps makes processes and meanings explicit [38, 47]. Focus codes were grouped into similar categories related to practices or challenges of person-centered mental health care, and constructed categories were compared with each other. Axial coding involved identifying relationships between the constructed categories and refining our themes. Sensitizing concepts on person-centeredness informed our axial coding process. Specifically, we regrouped our main themes into several broad domains that reflect key concepts in the person-centered care literature: [1] Patient as unique person, [2] Patient-provider relationships, [3] Sharing power and responsibility, [4] Connecting to family and community, and [5] Promoting person-centered care environments.

Data analysis was achieved through a team process that included the coding of an initial set of ten interviews by three team members (RA, MM, JB), followed by pairs of team members (RA, MM, JB, ME) parallel-coding the remaining interviews. Our data analysis team met monthly to discuss progress with coding, interpret and make sense of data, and consider implications for data collection (e.g., recruiting new informants, revising our interview guide). Emerging findings were also discussed at regular meetings with other team members (SD, JS, KM). The analysis process was collegial and drew on the different disciplinary perspectives of team members (social work, family medicine, psychiatry, health services, public health). Rigor and trustworthiness were established through prolonged engagement with the data, reflexive memo-writing, and team discussions. NVivo11 supported data management and analysis.

## Results

### Participant characteristics

We conducted 79 interviews with 65 FHT health professionals and administrators. Participants’ professional roles are presented in Table 1. Participants practiced within 18 FHTs spread across 9 health administrative regions of Ontario (covering the west, east, north, central and Toronto regions). Among the 18 FHTs, 11 were located in urban areas and 7 were in rural areas and they varied from having a smaller number of patients enrolled (< 8 000 patients, *N* = 6) to moderate (8001–20 000 patients, *N* = 6) or large numbers of patients (> 20 000 patients, *N* = 6).

**Table 1:** Professional roles of participants (*N* = 65)

Participant role	*N*
Social work	14
Family physician	11
Executive director	10
Mental health counselor	9
Psychiatrist	7
System navigator^1^	3
Nurse	2
Nurse practitioner	2
Occupational therapist	2
Psychologist	2
Program manager	1
Outreach worker^2^	1
Pharmacist	1

^1^ System navigators help service users navigate the health and community care system and connect to essential social services and community resources

^2^ Outreach workers engage service users in their communities (outside of clinical settings) and provide education and supports adapted to their needs

### Domains and themes

**Fig. 1:**
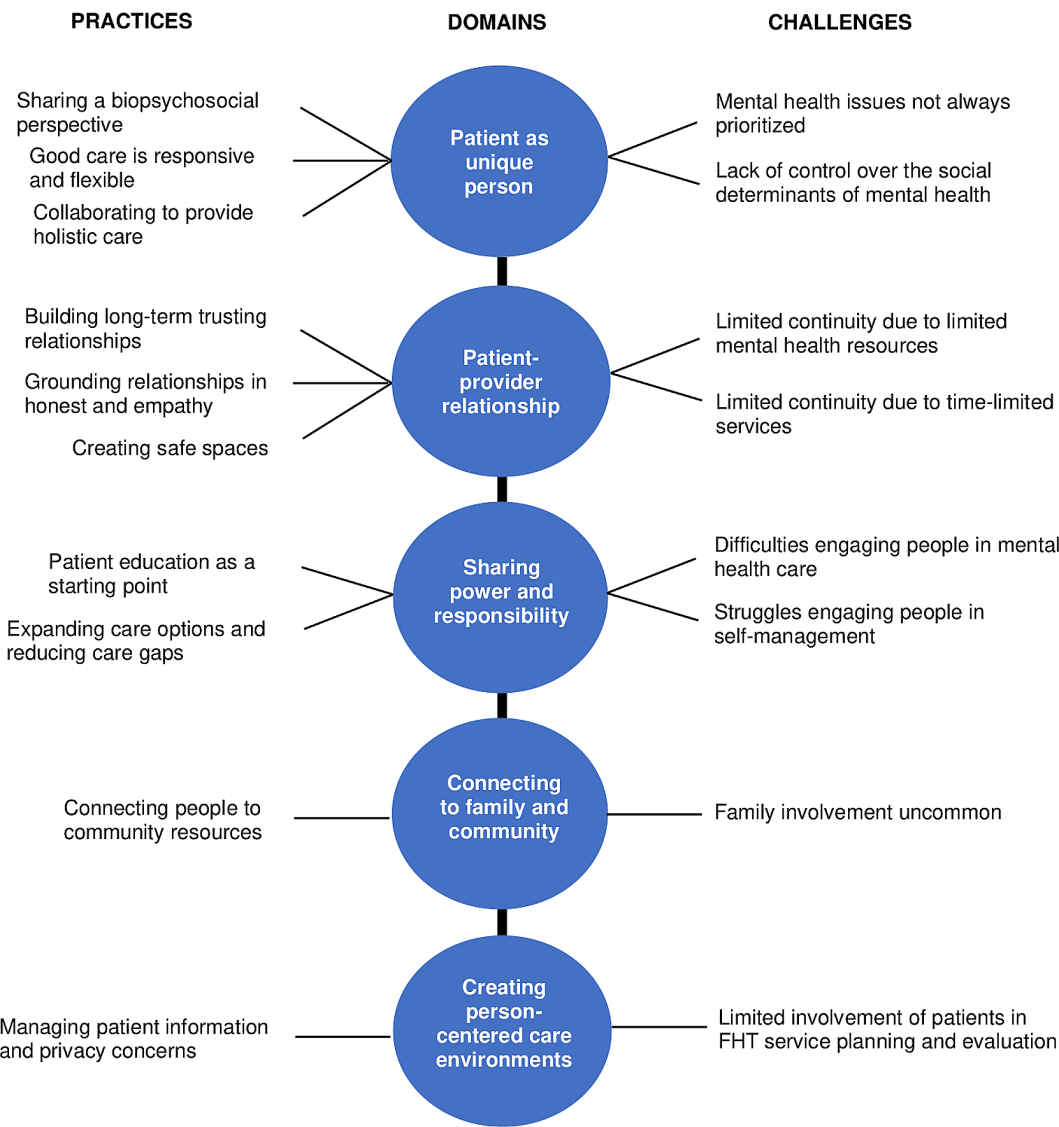
Visual summary of person-centeredness domains and themes

Themes related to perceived practices of person-centered care for common mental disorders are reported for each of the five conceptual domains deemed most relevant in our analyses. Perceived challenges in the delivery of care to patients with common mental disorders and their relationship to person-centered care are presented in Table 2. All themes are visually summarized in Fig. 1.

### Domain 1: patient as unique person

#### Sharing a biopsychosocial perspective

Participants emphasized that their role was not limited to managing a person’s mental or physical illness but rather to consider each person’s unique situation and psychosocial needs. This was a view widely shared within FHT teams, as much by physicians as other members of the interprofessional team. Participants recognized that psychosocial issues (e.g., family relationships, housing problems, employment issues or poverty) were “*prominent*” among people with common mental disorders and that these issues were sometimes at the root of their concerns.

Our social worker here is not surprised when she hears me think about mental health. She’s very used to hearing me, especially with diagnostic formulations, and looking at the social factors that drove this particular crisis or this relapse. (Physician 125)

Several participants mentioned that their patients would often present with multiple other non-mental health concerns, commonly related to their social context, highlighting the need to consider patients as ‘whole persons’ with multiple types of needs that required attention as opposed to seeing them simply as ‘patients with a disease’ requiring medical treatment for their mental illness.

When I ask the patient “What’s important to you?” or “If you could change one thing that matters to you right now, what would you say that is?” And 9 times out of 10, the response is not, “get the infection cleared in my leg” or it’s not “get my blood sugars within an A1C of point zero five”. (…) What matters to them is “I don’t have anybody in my life”, “My distance from my family”, “I can’t get to my appointments”, “I don’t know who to contact”, “I don’t know who these people are”, “I don’t have enough to eat”, “I’m worried about paying my bills.” You know, it’s those psychosocial things… (Systems Navigator 120).

…I don’t see people coming forward identifying to me that they’re depressed. They’re identifying that, you know, they need better housing. They can’t make ends meet or they’re angry about, just kind of like frustrated with their situation but they’re not coming in identifying as depressed and I’m certainly not talking to them as a depressed person. (Social Worker 206)

### Good care is responsive and flexible

FHT providers placed an importance on personalizing their care to meet an individual’s unique needs and circumstances. They most often used the term “*responsive*” to describe not only their ability to provide care in a timely manner but also adjust care to better meet a person’s particular needs. This was an active process of “*figuring out what they need*” by seeking information from the person, considering options and potential barriers to care, and finding the therapeutic approach that best suits the person. Being responsive included taking into consideration the person’s beliefs and preferences.

Other things are people’s personal preferences about different, and beliefs about different, the effectiveness of different therapies. So a patient who’s very skeptical about medication… We’ll sort of choose to not go down that route. (Physician 145)

Most participants expressed the belief that being flexible in their approach was necessary in FHT settings. This included being flexible with respect to the types of needs that they addressed as a priority and trying to ensure access to a broad range of treatments, care delivery modalities (e.g., in-person at the FHT, at home, via telehealth), and types of providers. Again, participants emphasized the need to find the approach that fit each person best.

Well, I guess to be flexible, because one treatment does not suit all. Like, CBT [cognitive behavioral therapy] is evidence-based, but it’s not going to treat everyone who walks in the door with anxiety and depression. I mean, I think it has a really good protocol to be able to follow, but that may not be the right fit for many. (Mental Health Counselor 220)

Being responsive also sometimes meant working outside the parameters of their normal role or the FHT’s usual working hours to meet individuals’ needs under more difficult circumstances. Several participants shared stories about how they went above and beyond for patients that were in a crisis situation, despite being in a position of professional liability or in what could be perceived as a boundary violation. Such examples were considered cases of “*good care*” that were supported by other members of the team.

### Collaborating to provide holistic care

Providing team-based care was widely viewed as central to the FHT identity, which was perceived as a facilitator to delivering holistic care. Physicians and mental health providers alike spoke about how it was routine within the FHT to “*look at the whole picture*” and “*deal with the whole person in many aspects*.” Addressing mental health needs as part of a holistic approach was facilitated by the co-location of mental health professionals within the FHT, which facilitated communication and teamwork.

So for them to be able to refer to other professionals who are working in collaboration, I’m sure has eased greatly the load for physicians and really improved the care overall for a patient because they access their primary care, but they can also access many different doors. And we, like I work very diligently with people, I let them know right away that we’re a team. So I want them to know who are the players on their team. If they’re seeing their doctor, a nurse practitioner and a nurse for different things, then I know right away who their team members are and I’m going to be collaborating with them in lots of different kinds of ways. (Social Worker 137)

### Domain 2: provider-patient relationships

#### Building long-term trusting relationships

Participants overwhelmingly viewed relationship-building as one of the main components of their work and a strength of the FHT model. As one mental health counselor stated, “*it’s all about the relationship*.” Providers, and especially family physicians and nurse practitioners, were in positions to develop long-term relationships lasting many years with their patients, which fostered trust among them. Providers’ knowledge of the persons in their care and the trust between them facilitated the detection of mental health problems and helped people feel more comfortable opening up.

And then I feel like most of patients now, they’ve been my patients for at least 10 years. So I kind of feel like I know them and I know what their normal state of mind is and so if they present with mental illness I’ll have an idea what they’re presenting with and what their normal personality is. (Physician 207)

I think people with mental health have trouble reaching out and admitting to them having an issue or concern and, you know, having that trusting relationship with them that they know they can approach you and that I can reach out to them when I feel that they need it as well. It’s kind of reading between the lines with them, and once you get to know them quite well, then you’re able to pick up on those cues. (Systems Navigator 227)

### Grounding relationships in honesty and empathy

Participants explained that it was important for them to be authentic and genuine in their relationships with people with common mental disorders and that this facilitated person-centered care. They also felt it was important to establish a standard of openness and honesty in their communications (e.g., “*being straight with them*”) and to interact with people with common mental disorders in a compassionate, non-judgemental, and empathetic way. One participant stated that it was often important to “*look at it from the patient’s point of view*” and “*put yourself in their shoes*”, a sentiment echoed by other participants.

### Creating safe spaces

Because of the stigma surrounding mental illness, one of the main challenges that participants faced was encouraging people with common mental disorders to disclose their symptoms and discuss their problems openly. Participants mentioned that the FHT setting made it easier for people to open up and receive mental health care.

When I came here, it was very clear to me that people are much more comfortable seeking out services in their family physician’s office because their family physician’s office is familiar and comfortable. Their family physician knows them better than anybody else in their life, most of the time. Their doc knows everything about them. And so, when they come here, it’s a familiar and comfortable place, so it’s not so onerous or scary or intimidating to go for mental health support. (Psychologist 224)

In addition to the familiarity of the setting, several participants mentioned that the key to creating safe spaces for patients was making time for those difficult conversations during consultations and really listening to patients.

It’s about listening. I think if a patient feels like they can trust you and that you’re only listening and not judging, they can open up. (Executive Director 212)

…I frequently hear back about the nurse practitioners and doctors like, “She really listens to me. She picked up on it. I didn’t really make the connection that that’s what was going on.” I do hear that feedback. The majority of people will really, really feel safe and heard by their primary care providers. They tell them things they normally would perhaps not. And like I said, our care providers are really good at probing for this. And because of the probing, the person will open up. But they feel safe. (Mental Health Counselor 107)

Having mental health providers co-located with medical staff within the FHT made warm hand-offs possible, further enhancing access to mental health care while ensuring the person’s comfort and sense of safety.

It’s helpful when the family doctor or the nurse practitioner would have somebody actually present for the appointment. And if I’m available they might say “Hey, you know, I think it would be a good idea to talk to [participant], I can bring her down, you could just say ‘hi’”. So I think that’s sometimes a helpful thing. It’s not as scary, they’ve already met me. (Social Worker 148)

### Domain 3: sharing power and responsibility

#### Patient education as a starting point

Several participants described patient education as being an important part of how they engaged patients early in their relationships with them. This included education around their mental health conditions and treatments but also around their role as professionals and how the team members work together to provide mental health care. According to one psychologist, “*people don’t know what psychology is*,* so you have to educate them*”, and this was echoed by other professionals (e.g., social workers, occupational therapists) who felt the need to explain their roles in mental health treatment to patients.

### Expanding care options and reducing care gaps

Participants described a range of services and treatment options that people with common mental disorders could access at the FHTs. This included medications but also psychotherapy, group therapies and workshops, as well as psychosocial services. As expressed by one social worker, “*it’s just about giving people choices*.” Expanding choices beyond drug treatments was seen as especially important, “*Not everybody wants to take drugs*,* not everybody can take drugs. We need something that we can offer to our patients*” (Nurse Practitioner 124). In some FHTs, providers active in scientific research actively sought to integrate new treatments in care for people with common mental disorders (e.g., neurofeedback) to close gaps in treatment in their communities.

### Domain 4: connecting to family and community

#### Connecting people to community resources

Participants perceived that a strength of their FHT was the connections made within their community, and people with common mental disorders were routinely referred to community resources that could help meet their needs. Several FHTs also had professionals working in formal roles as ‘System Navigators’ that were knowledgeable about community resources and that made linkages to those resources easier for team members and vulnerable patients.

If you took a look at that data, I think you’d see a dramatic, dramatic difference in how the patient reports quality of life. You know, and prevention of caregiver burnout… by linking them to the resources that can support them with their needs. It’s not that the navigator is doing all of that, but linking people to the resources that people by themselves don’t know how to access, or don’t know how to get in the door. Um or they’re too overwhelmed and exhausted and frustrated, and sick to put the work in. (Systems Navigator 120)

Patients did however sometimes experience barriers to accessing community services. Several participants described how important it was to advocate on behalf of people with common mental disorders, stating “*sometimes we have to push a little bit and try to get them in (to a service)*” (Mental Health Counselor 104) and “*patients fall down waitlists without advocacy*” (Nurse Practitioner 124).

### Domain 5: creating person-centered care environments

#### Managing patient information and privacy concerns

FHTs are interprofessional environments where team members share information and interact often, including electronically via their shared electronic medical records (EMR) system. Sharing an EMR allowed teams to work in a more efficient, coordinated way but it also meant that they had to routinely manage patients’ privacy concerns. Participants reported that they would often inform and reassure patients about how their personal information would be managed; however, they made it a point to explain to patients that information sharing was part of the team-based approach at the FHT.

Our notes are all in one place, right? So, doc, dietician, nurse practitioners can see what folks are talking about in counselling. And our clients know that, like I tell them when I’m first meeting with them, “Just so you know, there’s one central file, we’re all writing on the same thing. Anybody who is in contact with you will have access to those files.” (Mental Health Counselor 101).

Privacy concerns were viewed as particularly noticeable among people with common mental disorders, especially those living in smaller or rural communities where stigma remains a problem and anonymity is difficult to achieve.

**Table 2:** Challenges of providing person-centered mental health care

Domains and themes	Summary	Quotes
**Domain 1: Patient as unique person**		
Mental health issues not always prioritized	While the delivery of integrated mental health care was considered important within several FHTs, in other teams it was prioritized to a lesser extent. Some participants were critical of their FHT for mental health care being “not at the center” of their clinical programming or having patients’ mental health issues treated as an “afterthought.”	I think when there’s so many things going on, there’s frequently mental health stuff going on. But that’s kind of the last thing on the list that people are addressing. (Social Worker 123) I think part of the difficulty is, people come and they have like literally fifteen diagnoses and anxiety and depression just becomes another bullet. So, it’s not necessarily a forefront issue, but it is one of the things that impacts, I think, outcomes in ways that we’re not really clearly addressing or articulating. (Occupational Therapy 110) With our large mental health component, that mental health unfortunately is still kind of an afterthought. Even though if you were to speak to any of the family physicians, they would say that it’s probably one of the number one issues that they struggle with within their practice, and that they rely upon the most in terms of services, but most of the time we are talking about things like COPD, chronic heart disease…. (Social Worker 103)
Lack of control over the social determinants of mental health	The biopsychosocial perspective, while highly valued within teams, also led to feelings of frustration on the part of some providers. These participants felt that the impacts of their clinical work would always be limited given the social forces that influence health, and they shared a sense of helplessness with respect to being able to prevent mental disorders and promote mental health in a significant and sustainable way.	But it’s a drop in the bucket compared to what’s actually required to actually affect people’s lives in authentic ways. It has nothing to do with medicine. Nothing. It is all public health. It is all social work. It is all in society. It’s all, and it’s the built environment. It’s education, it’s housing, it’s income, it’s jobs, it’s employment, it’s gender, it’s immigration, it’s all of that stuff… That’s how you prevent people from having mental illness. (Physician 125) There are so many factors of living that are barriers for being able to make small changes, right? So many patients that I’m working with are either underemployed or working in jobs that are just horrible, so even to be able to take time off to come to the clinic, they’re losing money. (…) There may not be food in their home, or there may not be healthy food available, and they may not be sleeping because, you know, they’re having to choose between rent or heat, right? Like, there’s awful factors beyond our control… (Mental Health Counselor 220)
**Domain 2: Patient-provider relationship**		
Limited continuity due to limited mental health resources	Some participants reported that it was difficult for some patients to establish long-term relationships to support their mental health care when they were unable to establish a good relationship with the initial providers available to them. When “things don’t click” with the first provider available, there were not always other providers available to offer supports due to a lack of funding and limited mental health and FHT resources.	They’ll come back and say, “Okay, I’m doing better and the counselling is working” or “I don’t like the counsellor, I want someone different”, and how do we find that? How do we find that, especially because people don’t have resources, so if they don’t like the counsellor here, we don’t have a lot of options. (Physician 234) Like, there’s a lot of people that may not have a great relationship with their physician, or their mental health provider, or any of that, but that’s all you have. You let that go and you are out of luck. (Executive Director 219)
Limited continuity due to time-limited services	While services from FHT mental health providers are free of cost, they were not time unlimited. Several participants described how they were only normally allowed to offer a limited number of sessions to people seeking mental health care and that those people needing additional or longer-term supports were often oriented to other providers and services. This led to breakdowns in relational continuity for people in need of long-term mental health supports. This also put patients in a situation where they have to rebuild relationships with each new provider and retell their story each time, which was viewed as frustrating and a disincentive to seeking care.	Setting goals and having people come with the mindset that we have 8 sessions, so they use me, and that’s what I tell them. It’s like, “you have me for 8 sessions, use me. If this is a good time in your life where you can prioritize, you know, doing the work with me, then let’s go, and if it’s not a good time, then let’s delay it until it is a good time.” (Psychologist 224) But for somebody who struggles with a lifelong problem, often somebody seeing you three times as the crisis worker, I’m allowed to have three follow up visits with you. So it’s like boom boom boom, but then next time there’s a crisis which is 6 months from now. It’s a different crisis, could be a different crisis worker on the same team. And they’re allowed to see you three times, because they’ve got metrics, and they’ve got funding restraints. So those things, I find, you get a little bit and then you’re out. And then you’re back to me saying, “Well I don’t know what to do”. And then that happens over and over again. (Physician 116) We’ve heard patients say many times they just don’t want to keep retelling their story over and over again, and I think that’s something that’s lacking in the community is the continuity of care over a longer term. So I think from a patient’s standpoint, that’s something that’s sort of a disincentive for them to receive care because they don’t want to rehash their story, especially if they’ve been abused or something terrible has happened to them, it’s uncomfortable sometimes to share that, and I think having to share it with different people in different institutions at different times is very challenging for them. (Pharmacist 222)
**Domain 3: Sharing power and responsibility**		
Difficulties engaging people in mental health services	Most participants mentioned that engaging and involving people with common mental disorders in care was challenging. Several participants reported frustrations that their patients would sometimes miss their appointments (“no-shows”) or drop out of care despite services being free of cost. There was a belief among many participants that patients needed to be “ready” for care in order to remain engaged and benefit from treatment by FHT providers.	Everyone will say, there’s nowhere for long term mental health services. And I’m sure that they have a lot of the same sorts of frustrations that we do with having those resources and patients not showing. (Program Manager 111) Even when we offer it for free… Sometimes the uptake isn’t there. (Physician 116) A lot of people are… realize that they’re actually not ready. So back to the stages of change, where their stage of change are, when they start and when they actually finish are two different places. So they’re not comfortable with moving forward through those six weeks and therefore they drop out. (Mental health nurse 140)
Struggles engaging people in self-management	Most participants reported that it was challenging to work with people with common mental disorders and engage them in self-management, mainly because these illnesses affected patients’ level of motivation. Poor motivation was seen as the reason why some patients did not adopt healthier behaviours or complete self-help tasks prescribed by their providers, and why some patients failed to make clear and continuous progress during treatment. For some providers, receipt of services was contingent upon patients displaying the required level of motivation or ability to progress in treatment. Lack of progress in clinical recovery was a source of frustration for providers. Some providers expressed considerable frustration about how some patients seemed unable to take personal responsibility for their own mental health. This made the therapeutic alliance feel one-sided, rather than a shared responsibility.	I think we struggled with that, especially when we were having a lot of like chronic conditions, chronic disease, and with that was the, you know, we’d talk about self-management all of the time. Like, that’s sort of the big push in the literature, but we realize that self-management is really hard for people who have depression or anxiety. You know, it’s hard to be motivated to self-manage. (Occupational Therapist 110) I do need to see progress. I do like to see progress. I check in, see if people are ready or not. If they’re not doing a lot to motivate themselves by a certain point, I will say, “I think you need to come back when you are ready to engage” and will refer to the different services to meet the need right now. (Mental health worker 107) I don’t even know if it’s a Family Health Team issue or it’s just again the population, is movement in treatment requires motivation, the very nature of anxiety and depression is depleted motivation. So I personally find that the most difficult, is the very symptoms of the disease can act as barriers to successful treatment. (Mental health leader, 130) I think a lot of people do use physicians as resources because we are free to them and we’re accessible… but ultimately there has to be a way of empowering the person to make the change. Or at least recognize that there’s an issue and you know some of it is more severe and requires a medical approach. But a lot of it is behavioural and needs lifestyle coaching and improvement of self-esteem and all those other things that allow you to function well and feel good about yourself, which I can’t always empower people with. (Physician 116) It’s like they’re intelligent, they get it, they can apply the content, but when it’s happening in their lives every day, they don’t seem to be able to apply it to their own lives, and there’s this reliance that somebody else is going to do it for me, or that’s what therapy is for. I’ll just go tell my therapist about her. She’ll tell me what to do. So it’s like that personal responsibility I do get frustrated with sometimes. (Mental Health Counselor 223)
**Domain 4: Connecting to family and community**		
Family involvement uncommon	FHT providers recognized that mental health problems could be disruptive within families and that there were benefits to family engagement. However, most participants reported that they did not typically involve families directly in care planning or the treatment process. The exceptions to this were providers that provided direct care to families (e.g., family therapy) or to youth with mental health concerns. In the latter case, it was not uncommon to work in partnership with parents. Some participants suggested that recent changes to privacy legislation in Ontario were a major barrier to family involvement.	*Interviewer*: Are there circumstances where family would be included? Either in the care planning or in the treatment process? *Provider*: Uhm, not typically. (Psychologist 126) I don’t generally involve family members in the counselling. (Mental health counselor 101) I think it’s a challenge for the people who care about them. Because it’s, we don’t have a choice in that. There are lots of people that I believe that their intentions are admirable, they want to help their family member but legislation prevents us from including them. (…) Well, under the enhanced privacy legislation, it used to be we couldn’t give any information, but we could receive information. Now we can’t even receive information from a family member. So it’s really, it’s difficult and the messaging unfortunately that has to go out to patients is “unless it’s something I can act upon, please don’t tell me”. It’s a really untenable situation. (Mental health leader 130)
**Domain 5: Creating person-centered care environments**		
Limited involvement of patients in FHT service planning	Very few participants provided examples of people with common mental disorders or other patients being involved in service planning or decision-making at an organizational level within the FHT. Only one participant reported that her FHT used focus groups to consult patients but these were not well attended. The absence of this “patient voice” at an organizational level limited opportunities for patients to participate in shaping the person-centeredness of the FHT care environment.	I also sit on our, (quality improvement) committee here, so we’re trying go to get more patient participation, that’s not quite the right word. Patient engagement. What we’re really trying to… develop a patient engagement strategy. We have run a few focus groups to try and start that. But the patient voice isn’t particularly strong here, I don’t know what the patient voice is. I mean I would know one on one, but as a collective… I think that’s something as a Family Health Team we’re trying to work more towards… (…) …we invite people to focus groups. I think we had two focus groups, and one group had two people that came, and one maybe had six? And to each of them, twelve people said they were coming, and they just didn’t come. And we kind of do one more and we’ll kinda reassess where we’re going to go next with that… (Social Worker 123)

## Discussion

### Summary

This study sought to explore the perspectives and experiences of FHT providers related to the delivery of care to people with common mental disorders to shed light on the person-centeredness of this care. FHT providers perceived their care to be person-centered several ways. Teams adopted a biopsychosocial perspective and aimed to deliver care in a responsive, flexible manner that considered each person’s unique needs, preferences, and circumstances. They often worked interprofessionally to address mental, physical, and psychosocial needs in a holistic, whole-person approach. They valued building long-term relationships with their patients and the genuine, trusting, and empathetic nature of these relationships was considered essential in care for common mental disorders. In most cases, the FHT was the regular source of primary care for patients, and patients’ familiarity with the site and its providers was thought to facilitate mental health care by helping them feel comfortable, safe and less stigmatized. FHT providers provided people with information and access to a variety of treatments and services, aiming to provide them with as much choice as possible and reduce gaps in care. When additional mental health supports were needed, providers linked people to community resources that could provide those supports.

Yet, providers also reported several challenges to delivering person-centered mental health care (Table 2). These included practicing in settings in which mental health concerns were sometimes regarded as a lesser priority than physical health problems, witnessing gaps in continuity of care, struggling to engage and involve people with common mental disorders in services and the self-management of their conditions, facing barriers to family involvement, and the limited “patient voice” in FHT service planning and quality improvement activities. To our knowledge, this is the first qualitative study focusing on provider perspectives of person-centered mental health care in Canadian primary care settings. This is also one of the few studies focused on this topic in primary care settings generally, especially in team-based settings aligned to the medical home model.

### Comparison with existing literature

Previous studies on the quality of mental health care in primary care have found that providers strive to balance the need to address patients’ mental health concerns while adopting a holistic approach and addressing the full spectrum of their needs [6, 26, 48]. Our study extends this finding by illustrating how biopsychosocial and whole-person approaches were widely valued within FHT teams, thus laying a strong foundation for holistic, team-based care. Previous work has also shown that providers also recognize the importance of tailoring treatments and services to people’ unique needs and being flexible in their approach to mental health care [6, 34, 49]. Dobscha and colleagues described this as a process of discovery [6], consistent with FHT providers’ practices of “figuring out” their patients’ needs like a puzzle to solve. This individualization of care was facilitated by genuine, ongoing and trusting provider-patient relationships, and the centrality of these relationships to person-centered mental health care has been widely reported, both from the perspectives of providers [6, 27, 48, 50] and patients [26, 27, 33, 36, 49]. The relevance of empathetic listening and non-judgemental attitudes to creating the safe, comfortable conditions needed for conversations about mental health concerns has also been underscored [35, 49, 50]. Finally, in FHTs as in other primary care settings, the presence of mental health professionals working in a co-located model of care not only expands treatment options and patient choice but also facilitates care coordination and patients’ ability to navigate and connect to other helpful resources in the community [28, 51].

Our study also builds on and extends previous work revealing important challenges faced by FHT providers, notably in their ability to engage people with common mental disorders in FHT services and involve them as partners in their care. In particular, we found that providers experienced frustrations about patients missing opportunities to receive care, missing appointments, and dropping out of treatment, consistent with other studies describing problems of disengagement from mental health services in primary care and community settings [52, 53]. People with common mental disorders want to share responsibility over their care with providers and participate in care decisions and planning [54], but this has been shown to be poorly implemented in primary care [24, 54, 55]. Previous studies have also revealed that providers tend to attribute the causes of poor engagement and involvement to factors external to them, including patients’ disorganization or lack of insight, language or cultural barriers, and societal stigma [52, 55]. In the current study, the problems of engagement and involvement were laid mostly at the feet of patients, who were seen as often lacking readiness or motivation. Some providers expressed frustrations when patients seemed unable to take responsibility for their own self-care and recovery. This contrasts with results from Dobscha and colleagues, who found that providers practicing in a person-centered approach were sometimes worried about giving patients *too much* responsibility, putting them in the position to feel overwhelmed or discouraged if a care plan failed [6]. Providers in our study also did not routinely engage service users’ families in the care they provided, though they were open to doing this in some circumstances. These findings are consistent with other studies illustrating the apparent complexity of working with families and involving them in mental health care [56, 57]. Our findings highlight a clear need for investments in training and supports for engaging patients and families in the mental health care delivered within FHTs.

The perceived challenges related to continuity and prioritization of care for common mental disorders have similarly been observed elsewhere, though this remains understudied in primary care settings. Breakdowns of relational continuity (when provider-patient relationships did not “click” or when time-limited services ended) have previously been reported [26, 33, 58], as have problems of informational continuity (communication problems and concerns about patients having to retell their stories to multiple providers) [58, 59]. Issues of management continuity have also been raised in the literature [58] but may have been less problematic in the current study given the co-location of mental health providers within the FHT and shared EMR systems. Continuity of care issues were seen as stemming from a lack of government funding for mental health resources, a situation echoed and deplored by other authors [6, 34, 37, 60]. Such problems and the lack of priority given to mental health care within FHTs and the broader system may also reflect the limited role that people with mental health concerns have historically played in service planning and improvement in Ontario and other jurisdictions [61–63].

### Strengths and limitations

A major strength of our study was the number and types of perspectives that were included in our study sample. Studies investigating the quality of mental health care in primary care from the perspective of providers have often relied on a single perspective (e.g., family physicians) whereas we interviewed FHT administrators and a diverse group of health professionals. This diversity and the number of interviews we conducted provided a richness to our dataset and lends confidence to the findings we reported. Another strength was our focus on services delivered within the FHT model and resulting ability to explore the way team dynamics contributed to the practice of person-centered mental health care. Team-based care is central to both primary care and mental health care and our study highlights how the values, practices and challenges of teams can help or hinder person-centeredness.

At the same time, focusing on FHTs may limit the transferability of our findings to other clinical contexts within and outside Ontario. FHTs are not the only type of primary care clinics aligned to the medical home model in Canada thus how mental health services are structured and integrated within them may differ. Another limitation of the study relates to the recent changes in Ontario’s service and policy contexts. This includes service transformations sparked by the COVID-19 pandemic, such as changes to providers’ scopes of practice and the growth in virtual care. Recent work by our team found that the pandemic and its consequences placed major strains on FHT providers and may have impacted person-centered mental health care in multiple ways, such as by improving patient service engagement through telehealth but impeding providers’ ability to establish or maintain therapeutic relationships with patients [64]. In addition, recent health reforms in Ontario were also introduced with an aim to strengthen person-centered care through the creation of Ontario Health Teams, groups of organizations providing an integrated continuum of care to communities across the province [65]. FHTs have begun joining these Teams, thus strengthening their ties to acute, long-term and home care providers in their regions. However, no evidence to date suggests that the above service and policy changes have significantly changed providers’ practices of person-centered mental health care in primary care and we remain confident that our findings are relevant and actionable given their consistency with other studies. Still, further research to examine the possibility of recent shifts in practices related to person-centered mental health care in Canadian and international contexts seems warranted.

### Implications for practice and policy

According to the College of Family Physicians of Canada, a core pillar of the Patient’s Medical Home model is care that it is patient- and family-partnered [12]. This includes strategies that FHTs have already largely adopted, such as delivering a range of care options beyond the traditional office visit and providing personalized care that is responsive to patients’ needs and preferences. However, our study suggests that other strategies of this pillar are not fully in place, notably to involve patients and families in shared decision-making processes, support self-management for each patient, and involve patients in FHT’s ongoing planning and quality improvement activities [12]. These three strategies seem ideal as targets for quality improvement efforts that could enhance the experiences of people with common mental disorders in their care. Interventions that target patients’ readiness and motivation, such as motivational interviewing, should be more widely practiced to reduce barriers to care and involvement [66, 67]. We further endorse the College’s position that mental health services in the Patient’s Medical Home should be empowering, strengths-based, and foster hope, consistent with a recovery-oriented approach [14]. Finally, our study findings also highlight the relevance of a ‘whole of society’ approach mental health policy for Ontario. Providers recognized the important influence that social factors have on the mental health of FHT patients. This calls for a greater focus on social interventions in primary care [68] as well as intersectoral and public health actions that address upstream social determinants, reduce inequities, and promote sustainable population mental health and well-being [69, 70].

## Conclusion

Family Health Teams provide comprehensive, team-based primary care services to communities across Ontario aligned to the Patient-Centered Medical Home model. However, if these clinics are to achieve this vision, they must deliver integrated, person-centered mental health services as a core element of their design [71]. Our study suggests that many FHTs have built a strong foundation of person-centered practices for people with common mental disorders but that additional strategies should be implemented to improve care experiences and adhere more closely to recent medical home models.

### Electronic supplementary material

Below is the link to the electronic supplementary material.

Supplementary Material 1

## Data Availability

The data used for this study are available upon reasonable request to the corresponding author.
