# Supplementary material for: Person-centered care for common mental disorders in Ontario’s primary care patient-centered medical homes: a qualitative study of provider perspectives
Source: BMC Prim Care. 2024 Aug 2;25:278. doi: 10.1186/s12875-024-02519-w (PMC11295484; doi:10.1186/s12875-024-02519-w)
Supplement: Supplementary file 1 — Supplementary Material 1 [file 12875_2024_2519_MOESM1_ESM.docx]

### Interview Guide*

### Project title: Incentives and disincentives for treating depression and anxiety in Ontario Family Health Teams

### Background statement:

### This aim of this interview is to explore the factors that make it more or less likely for people with depression or anxiety disorders to receive high-quality care in Family Health Teams (FHTs). We’ll be exploring various aspects of care quality and we’re interested in your point of view and experiences on these topics.

### As mentioned in the consent form, you should feel free to speak openly given that our conversation will remain confidential and measures will be taken to protect your anonymity. The interview should last approximately 60 to 90 minutes but do not hesitate to end the interview at any time.

### Do you have any questions before beginning?

### Participant characteristics:

### What is your profession and role within the FHT?

### How long have you worked at this FHT?

### How many years of experience do you have in your profession?

**Definitions of incentives and disincentives:**

We’re now going to talk about the incentives and disincentives that influence the care received by people with depression or anxiety disorders. Remember that incentives are things that encourage a certain action (make you want to do something) and disincentives are things that dissuade or deter a certain action (make you less likely to do something). This study is exploring the wide variety of incentives and disincentives that may exist including financial as well as non-financial.

Do you have any questions before we continue?

**Domains:**

- Technical care
- Access
- Equity
- Structural quality
- Person-centeredness
- Efficiency
- Other

**Topic – Person-centeredness:**

What are the incentives/disincentives for delivering person-centered care to people with common mental disorders?

Potential prompts:

- Involving patients in care decisions or care planning
- Involving patients’ families in care
- Providing patients with self-management supports
- Facilitators and challenges of delivering person-centered care

* This guide will be adapted depending on the type of participant (e.g. professional vs team director vs policymaker, etc.) and will evolve as the study progresses and information on new categories and relationships between categories of the theoretical model are sought.
